# Designing for the Intersection of Aging and Disability: Application of the TechSAge Technology Intervention Model

**DOI:** 10.1093/geroni/igaf122.978

**Published:** 2025-12-31

**Authors:** Laura Rice, Tracy Mitzner, Jon Sanford, Elena Remillard, Wendy Rogers

**Affiliations:** University of Illinois College of Applied Health Sciences, Urbana, Illinois, United States; Person in Design, Atlanta, Georgia, United States; Georgia State University, Atlanta, Georgia, United States; Georgia Institute of Technology, Atlanta, Georgia, United States; University of Illinois Urbana-Champaign, Champaign, Illinois, United States

## Abstract

As people live longer with disabilities acquired early in life, the additive effects of aging create unique challenges at the intersection of aging and disability. Technology interventions can minimize barriers and create facilitators to support performance of activities integral to health and quality of life. The absence of a theoretical framework to guide such interventions, in either gerontology or rehabilitation, created gaps in the knowledge base required to meet the needs of these individuals. We proposed the TechSAge Technology Intervention Model (TechSAge-TIM) to support activity engagement of older adults aging with long-term disabilities through technology design that bridges the gap between intrinsic capabilities and functional abilities (Mitzner et al., 2018). We have since utilized the model to advance understanding of technology-based supports for persons aging with long-term mobility, hearing, and vision disabilities. We describe herein applications involving people with mobility disabilities. We identified unmet needs by exploring lived experiences and used the TechSAge-TIM to guide research and development of a seated tele tai chi program for exercise/social engagement, smart bathroom technologies, and an automatic fall detection system for wheelchair users. These applications advanced the field of aging and disability and provided a roadmap for future research and development efforts.

